# An Electrostatically Preferred Lateral Orientation of SNARE Complex Suggests Novel Mechanisms for Driving Membrane Fusion

**DOI:** 10.1371/journal.pone.0008900

**Published:** 2010-01-26

**Authors:** Ting Guo, Lin-Chen Gong, Sen-Fang Sui

**Affiliations:** 1 State Key Laboratory of Biomembrane and Membrane Biotechnology, Center for Structural Biology, School of Life Sciences, Tsinghua University, Beijing, China; 2 Department of Physics and Center for Advanced Study, Tsinghua University, Beijing, China; Institute of Biophysics and Nanosystems Research (IBN), Austria

## Abstract

Biological membrane fusion is a basic cellular process catalyzed by SNARE proteins and additional auxiliary factors. Yet, the critical mechanistic details of SNARE-catalyzed membrane fusion are poorly understood, especially during rapid synaptic transmission. Here, we systematically assessed the electrostatic forces between SNARE complex, auxiliary proteins and fusing membranes by the nonlinear Poisson-Boltzmann equation using explicit models of membranes and proteins. We found that a previously unrecognized, structurally preferred and energetically highly favorable lateral orientation exists for the SNARE complex between fusing membranes. This preferred orientation immediately suggests a novel and simple synaptotagmin-dependent mechanistic trigger of membrane fusion. Moreover, electrostatic interactions between membranes, SNARE complex, and auxiliary proteins appear to orchestrate a series of membrane curvature events that set the stage for rapid synaptic vesicle fusion. Together, our electrostatic analyses of SNAREs and their regulatory factors suggest unexpected and potentially novel mechanisms for eukaryotic membrane fusion proteins.

## Introduction

Biological membrane fusion is a basic cellular process necessary for exocytosis, endocytosis and exchange of vesicular contents in eukaryotic cells. Fusion of membranes is highly energetically demanding since tremendous electrostatic repulsion between negatively charged lipid bilayers has to be overcome.[1] *In vivo*, fusion is catalyzed by three families of conserved proteins collectively termed the SNARE proteins (soluble N-ethyl-maleimide-sensitive factor attachment receptor).[1], [2], [3], [4] During membrane fusion, SNARE proteins, anchored on the two fusing membranes, combine to form SNARE complex, a highly stable coiled coil structure consisting of four α-helices. Formation of the SNARE complex is thought to supply the energy needed to drive close apposition of fusing membranes and perhaps initiate the fusion process.[5] In addition, at least two auxiliary proteins, complexin and synaptotagmin, also participate in membrane fusion and are particularly important for rapid and tight control of fusion at synapses in response to neuronal activity.[6], [7]

The transient and critical molecular events during which SNAREs and auxiliary proteins control membrane fusion are still poorly understood. One reason is that, compared to extensive gain- and loss-of-function studies of the roles of fusion proteins, quantitative understanding of electrostatic and other physical interactions between membranes, SNARE and auxiliary proteins has been lacking. For instance, the synaptic SNARE core complex possesses a cluster of positively charged residues near its C-terminus.[8] For years, these positive charges have been postulated to neutralize electrostatic repulsions between fusing membranes and promote their close contact.[8], [9], [10] However, is such an effect quantitatively plausible? Besides these charges, what are the contributions of other charges on the surface of the SNARE complex? Moreover, how will recruitment of complexin and synatotagmin rearrange the electrostatic properties of SNARE complex? Could the electrostatic potential of SNARE and auxiliary proteins induce bending of fusing membranes, an essential step towards fusion[11], [12]? Questions such as these cannot be addressed by qualitative speculations, but their answers will likely provide important mechanistic insights into the basis of biologically catalyzed membrane fusion, an evolutionarily innovation key to the emergence of eukaryotic life.

A predominant form of protein-membrane physical interactions during biological membrane fusion is electrostatic interactions. During fusion, the SNARE complex is positioned close to and sandwiched between two fusing membranes. At such a distance, electrostatic forces overcome ionic screening effect and will have a major influence on interactions between molecules.[13] For example, the electrostatic force born by lipid bilayers at this stage will partially determine the geometry of fusing membranes and is thus critical for understanding the mechanisms of fusion. The magnitudes of electrostatic interactions between proteins and membranes can be assessed by solving the nonlinear Poisson-Boltzmann equation for large supramolecular systems using techniques developed over the past two decades.[13], [14] This method is now widely in use[15], [16], [17], [18], [19] and yields highly precise protein-membrane interaction free energies when compared with experimentally determined results.[20], [21] Meanwhile, enabling the calculations for the membrane fusion system, crystal structures for SNARE complex, synaptotagmin and a SNARE/complexin complex have been solved;[8], [22], [23], [24] the structural models of SNARE/synaptotagmin complex has also been reported and experimentally tested.[9], [10]

Here, we analyzed intermolecular electrostatic interactions in SNARE-mediated membrane fusion by solving nonlinear Poisson-Boltzmann equation on all-atom models of the system. We focused on interactions between fusion proteins and membranes, but not the energetics of fusion of pure lipid systems which has been studied in many other works (e.g.,[11], [25], [26], [27]). Several interesting predictions were made from this study. We found from both structural constraints and energetic calculations that SNARE complex exhibits a preferred lateral orientation between fusing membranes. The existence of such a preferred orientation implies a novel “propeller” mechanism for SNARE complex to directly drive membrane fusion. Moreover, at this preferred rotation, the electrostatics of SNARE complex interacts with negatively charged fusing membranes such that electrostatic repulsions likely promote bending of the fusing membranes. We also found that both complexin and synaptotagmin dramatically rearrange the pattern of surface electrostatic potential of SNARE complex. The scenario that emerges from these predictions is consistent with previous studies (e.g., [28]). Taken together, these analyses suggest additional layers of mechanistic control of membrane fusion by eukaryotic fusion proteins.

## Results

### A Putative Preferred Lateral Orientation for SNARE Complex between Membranes

**Figure 1:**
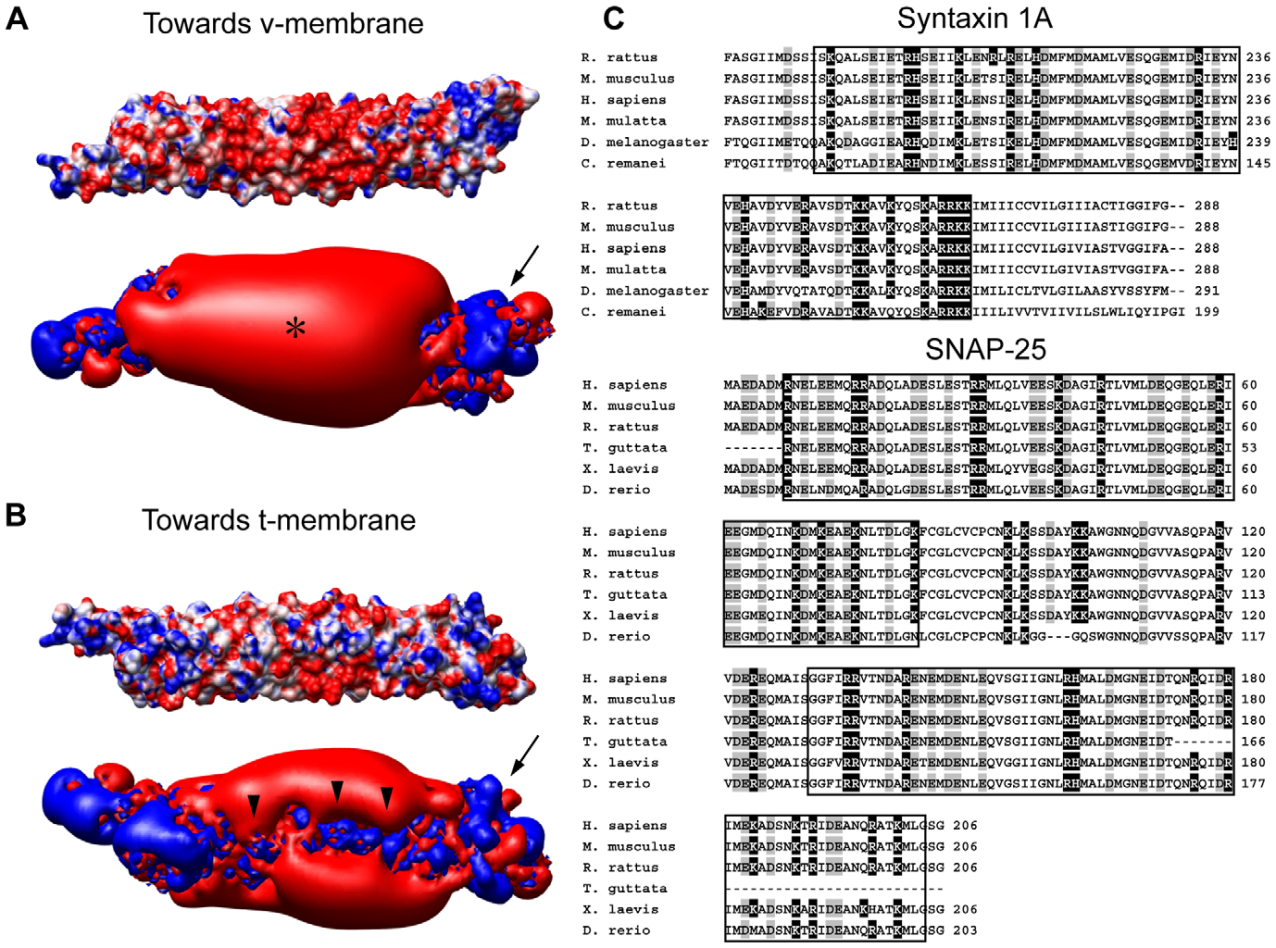
Electrostatic potential profile of the SNARE complex. (A) and (B) Patterns of surface electrostatic potential of the neuronal SNARE complex colored to molecular surfaces at ±7 kT/e (top panels) or to ±1 kT/e isopotential contours at (bottom panels). Positive potential is colored in blue, and negative potential is in red. Asterisk: the bulk of negative charges facing the v-membrane. Arrows: positive charges enriched in C-terminus of the SNARE complex. Arrowheads: positive charges selectively localized on the side facing the t-membrane. (C) Multiple alignment of SNAP-25 and syntaxin protein sequences across different species. Negatively charged residues are shaded in gray, and positively charged residues are shaded in black. The SNARE motif is framed in boxes.

We first constructed surface potential profiles for the synaptic SNARE complex based on its crystal structure.[8] First, we confirmed that there are significant positive charges in its C-terminus (Fig. 1A and 1B, arrows), a feature previously suggested to help neutralize repulsions between negatively charged lipid headgroups of opposing membranes.[8], [10], [23] Interestingly, by contrast, the rest of SNARE complex shows mostly negative electrostatic potential but will also lie close to the membranes before fusion (Fig. 1A, asterisk). This raises the question of whether electrostatic charges of SNARE complex simply facilitate or inhibit the approaching of fusing membranes.

**Figure 2:**
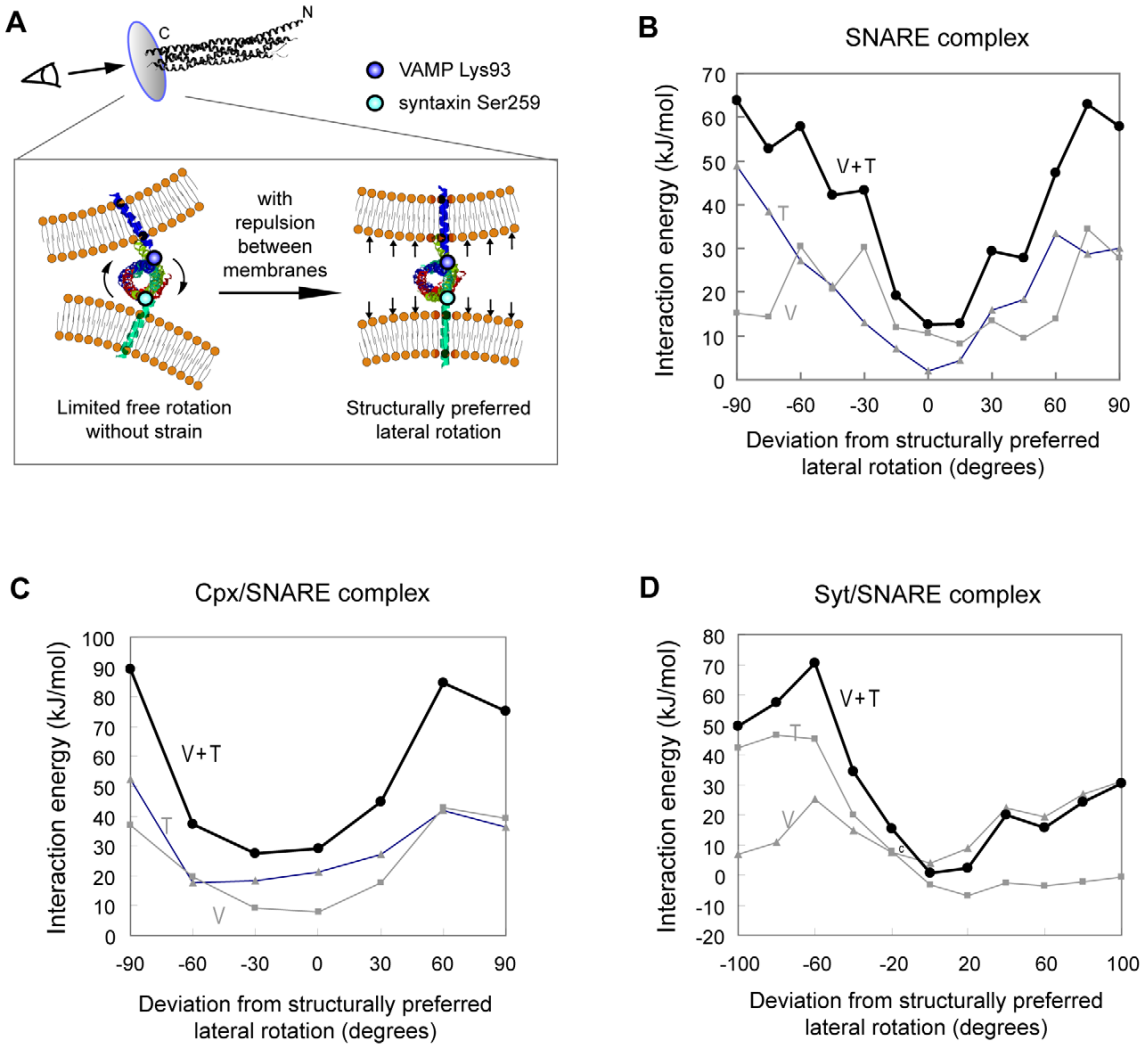
The SNARE complex has a structurally and energetically preferred relative orientation between fusing membranes. (A) Schematic of the structurally preferred orientation. Linker regions between the SNARE motif and TMDs are sufficiently short (∼7.5 Å and ∼0 Å) compared to the SNARE motif superhelix (which has a periodicity of ∼60 Å) and are α-helical continuation from it. During fusion, repulsion between opposing membranes aligns the VAMP and syntaxin TMDs roughly orthogonal to membrane surfaces. This thus aligns the Lys93-Ser259 axis also in the same line. Lys93 and Ser259 are the C-terminal ends of the SNARE motif in VAMP and syntaxin. (B) – (D) The energetically most favorable orientation exactly matches the structurally preferred orientation identified in (A). Interaction energies between two fusing membranes and (B) the SNARE complex, (C) the complexin/SNARE complex and (D) the synaptotagmin/SNARE complex were calculated at various orientations. V: SNARE's interaction free energy with a 40 nm-diameter vesicular membrane representing a typical synaptic vesicle. T: SNARE's interaction energies with a planar target membrane. V + T: sum of interaction energies with the v- and t-membranes.

To functionally dissect SNARE's electrostatic interactions with the membranes, we first asked whether there is a preferred lateral orientation of SNARE complex between fusing membranes. Interestingly, we found that both intrinsic structural constraints and intermolecular interactions point to a single preferred rotation for SNARE complex prior to fusion (Fig. 2). Structurally, after its assembly, the SNARE core complex will not be allowed to laterally rotate freely because the C-terminus of the core complex continues in α-helix immediately into the transmembrane domains (TMDs), which are fixed in the two opposing membranes (Fig. 2A). In the neuronal SNARE complex, the linkers joining SNARE motifs to the TMDs are short (0 amino acid residues or ∼0 Å for VAMP, and ∼5 residues or 7.5 Å for syntaxin); in addition, the SNARE core complex and TMDs are within a continuous α-helix.[29], [30] Meanwhile, prior to membrane fusion, the membranes repel each other by electrostatic forces but are held together by SNARE TMDs. As the result, the TMDs of SNARE, and thus the C-terminal ends of SNARE core complex, are aligned roughly orthogonal to the fusing membranes (Fig. 2A). Thus, considering that the SNARE complex superhelix has a periodicity of ∼60 Å, very limited freedom of lateral rotation (around one tenth of the periodicity or ∼36°) is allowed even if the linker is free to rotate. Since the terminal ends of the SNARE motif in syntaxin and VAMP are Ser259 and Lys93, we could identify the structurally preferred orientation of SNARE by aligning the Ser259-Lys93 axis orthogonal to fusing membranes.

To our surprise, the structurally preferred orientation exactly matches the orientation where SNARE complex exhibits minimal electrostatic interaction energies with fusing membranes (Fig. 2B). We initially placed SNARE core complex between model v- and t-membranes and calculated its interaction free energies with the two membranes at different lateral orientations (see below and Methods for details). We found that the most energetically favorable orientation is actually the orientation determined by structural constraints (Fig. 2(B)). Even a 30° deviation from the structurally preferred orientation incurs punishingly high electrostatic repulsion (∼30 kJ/mol) from neighboring membranes. Moreover, this is true not only in neuronal SNARE complex but also in complexin/SNARE and synatotagmin/SNARE complexes (Fig. 2C and 2D). In addition, charged residues of the SNARE core complex are highly conserved (Fig. 1C). These independent lines of evidence together strongly support the existence of a previously unrecognized preferred orientation for SNARE complex between membranes. Thus, the unique patterns of surface charges of SNARE complex and regulatory factors appear to cooperate with intrinsic structural constraints to ensure a preferred orientation for SNARE complex relative to fusing membranes.

This putative preferred orientation immediately suggests a simple and novel “propeller” mechanism for SNARE to directly drive fast membrane fusion. Synaptotagmin, an important auxiliary fusion protein and the putative Ca^2+^ sensor during neuronal transmission, can bind to both membrane and the SNARE complex during membrane fusion.[31], [32] Remarkably, at the preferred orientation of SNARE complex that we identified, Ca^2+^-binding loop in synaptotagmin C2B domain points straight toward the v-membrane in the synaptotagmin/SNARE complex[9]. This Ca^2+^-binding loop is the motif that will insert into the membrane in response to Ca^2+^ influx, the signal that triggers synaptic vesicle release.[31], [32], [33] Thus, for any fusion-ready vesicle, if some of its synaptotagmin/SNARE complexes have their Ca^2+^-binding loop insert into the opposite t-membrane in response to calcium influx, the result will be turning over of the entire SNARE complex by 180° away from its resting preferred orientation. During this process, the TMDs of SNARE proteins are expected to act as a propeller and drastically distort local membrane structure, potentially enabling fusion pore formation. Indeed, membrane insertion of C2B occurs preferentially towards the PIP_2_-rich microdomains of the t-membrane.[34], [35], [36] We propose that this model provides an energetically plausible means to instantaneously promote fusion (see Discussion).

### The SNARE Complex Electrostatically Repels v-Membrane Strongly but Interacts with T-Membrane Weakly

Following the identification of a structurally determined and energetically most favorable orientation, we analyzed the two sides of SNARE complex that face the v- and t-membranes, respectively, by following this putative preferred orientation. In the side facing the v-membrane (i.e. the membrane that bears the TMD of VAMP), surface electrostatic potential is mostly negative (Fig. 1A). By contrast, basic residues are enriched specifically throughout the side facing the t-membrane (Fig. 1B, arrowheads). Hence, SNARE's electrostatic interactions with the v- and t-membranes are likely to be of very different nature.

**Figure 3:**
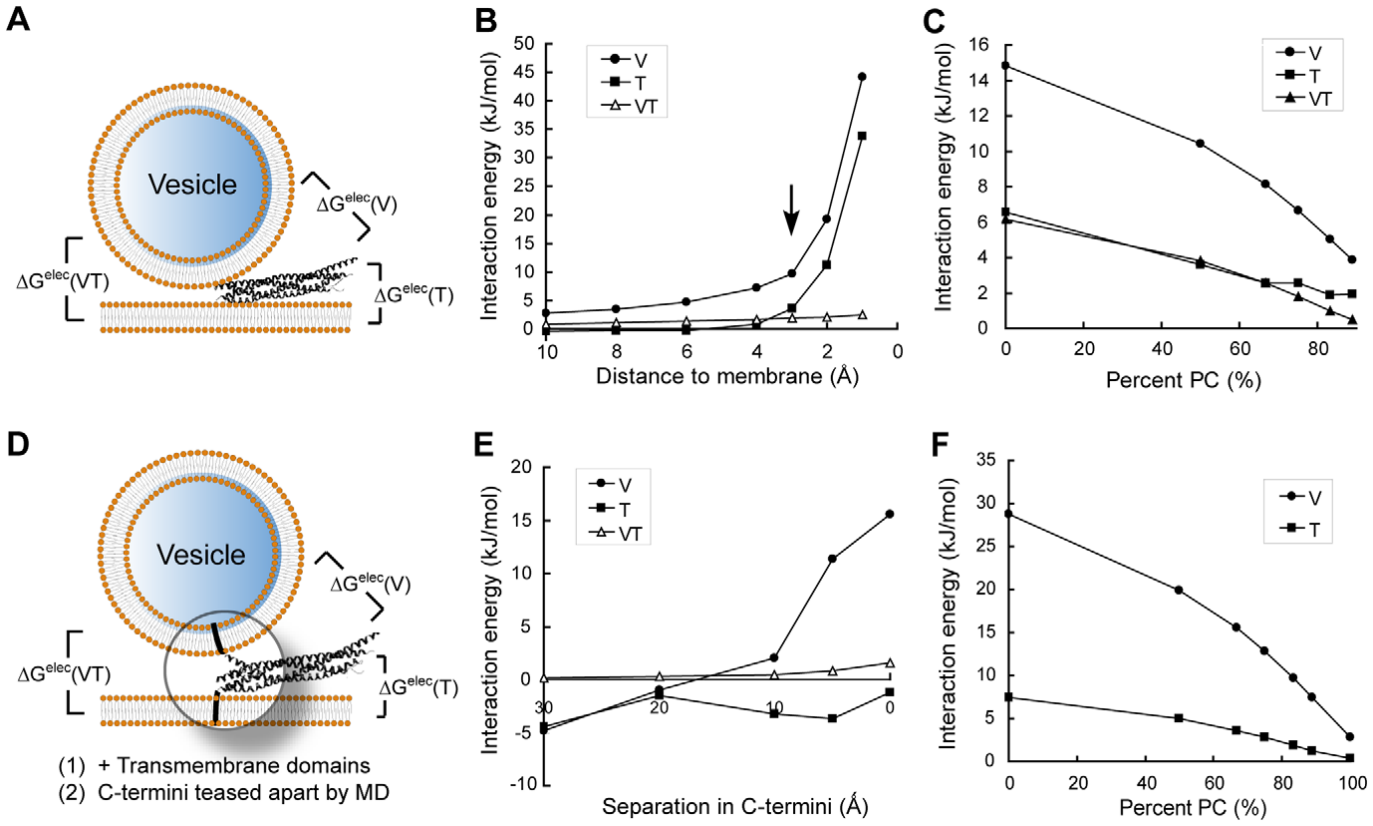
Electrostatic interaction energies between the SNARE complex and fusing membranes. (A) Schematic diagram and definitions of intermolecular interaction energies presented in (B) and (C). In (A) through (C), only the SNARE core complex is considered. Interaction free energies are calculated for (B) a series of SNARE/membrane distances and (C) different membrane lipid compositions. Arrow in (B) indicates the most physiologically relevant distance when the closest points between SNARE and membranes are 3 Å, the thickness of a layer of water.[19] In (D) through (F), TMDs of VAMP and syntaxin are present and embedded in membranes. Furthermore, the C-terminus of the SNARE motif is partially unraveled into individual α-helices by molecular dynamics simulations to represent trans-SNARE complex. Interaction free energies are then calculated for (E) a series of SNARE motif C-terminus separation distances and (F) different lipid compositions of the membranes. Conclusions drawn from both groups of studies are essentially the same. V (circles): Interaction energies between the SNARE complex and the v-membrane. T (squares): Interaction energies between SNARE and the t-membrane. VT (triangles): Interaction energies between the v- and the t-membranes if the SNARE complex were extracted.

To quantitatively characterize electrostatic interactions, we determined the levels of interaction free energies between SNARE complex and membranes using the nonlinear Poisson-Boltzmann method (Fig. 3A).[15], [16], [17], [18], [19], [20] It has been shown that magnitudes of electrostatic interactions between protein and membranes derived with this method agree very well with experimentally determined free energies of interaction.[20]

We first calculated interaction free energies between the SNARE core complex and atomic-detail model membranes at a series of protein/membrane distances (Fig. 3B). We built models of the SNARE/v-/t-membrane ternary complex based on current understanding of the fusion-competent state. Models representing the v-membranes were bent to a series of degrees to reflect spontaneous curvatures of vesicles. The membranes are comprised of 2∶1 PC/PS, a lipid composition commonly used to simulate the electrostatics of biological membranes.[15], [17], [18], [19] Consistent with electrostatic profiles (Fig. 1A), we found that the SNARE complex as a whole repels the v-membrane strongly (Fig. 3B). In particular, electrostatic repulsion between neuronal SNARE complex and a typical synaptic vesicle of a 40 nm-diameter is ∼10 kJ/mol, a significant level considering that the free energy required for fusion to occur is ∼40 kJ/mol.[37] 10 kJ/mol of repulsion energy occurs at a most physiologically relevant distance when the two molecules are close enough and yet dehydration has not yet occurred to each of them.[19] Such repulsion, together with antagonizing forces from the TMDs, could in principle generate membrane bending (see below).

On the other hand, the SNARE complex has very weak electrostatic interaction with the t-membrane, consistent with surface potential patterns (Fig. 1A). Interaction energy between SNARE and the t-membrane is nearly 0 kJ/mol over a wide range of intermolecular distances (Fig. 3B). This may ensure close apposition of fusing membranes at a relatively low energy cost given that SNARE repels the v-membrane electrostatically.

We next varied the lipid composition of model membranes and repeated the calculations (Fig. 3C).[19] Because PC carries no net charge in its headgroup, mixing in more PC leads to decreased levels of electrostatic repulsion as expected. However, observations made on 2∶1 PC/PS membranes are qualitatively similar to what is found in a wide range of lipid compositions (Fig. 3C).

Because some models suggest that SNARE complex assembly is not completed before the onset of fusion,[38] and because TMDs are not included in the crystal structures, we further added SNARE TMDs to the crystal structure and performed molecular dynamics simulations to partially unravel SNARE complex C-terminus. This gave us a structural model for “trans-SNARE complex”, a putative fusion intermediate in the SNARE assembly pathway (Fig. 3D). Manipulations to unravel the coiled coil are relatively straightforward due to the simple helical secondary structures of individual SNARE helices, short juxtamembrane regions, and helical continuity between SNARE core helices and TMDs.[29], [30] The trans-SNARE complex was first embedded in the t-membrane to calculate its interaction energy with the v-membrane. Next, this process was reversed to calculate the interaction energy between trans-SNARE complex and the t-membrane. We used implicit lipid bilayers here in order for TMD embedding.[39], [40] We compared interaction free energies obtained from explicit (atomic-detail) and implicit membranes using SNARE core complex, and found their differences to be <3 kJ/mol in all cases tested.

In the first set of calculations (Fig. 3E), the C-terminus of trans-SNARE complex was opened up by a series of distances up to 30 Å, which represents the thickness of a lipid bilayer. Intriguingly, the energy landscape of fusion computed by this method is mostly the same as that from Fig. 3A. We also tested this model by using a series of different membrane charge densities, and again, similar interaction patterns were observed (Fig. 3F). Together, we conclude that the SNARE complex shows strong electrostatic repulsion to the v-membrane, and very weak electrostatic interaction with the t-membrane.

**Figure 4:**
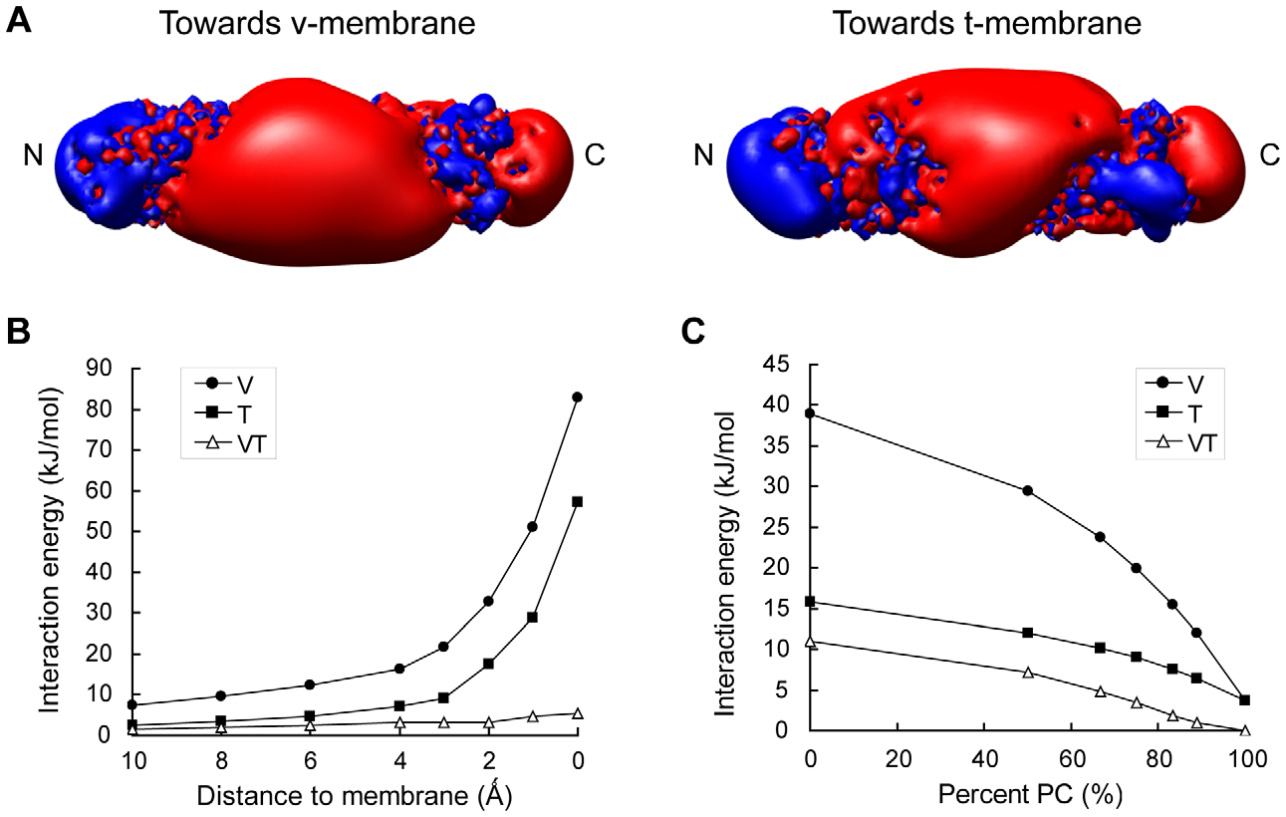
Electrostatic properties of the endosomal SNARE complex. (A) Surface electrostatic isocontours for the endosomal SNARE complex at ±1 kT/e. Positive potential is colored in blue, and negative potential is in red. Interaction free energies between SNARE complex and fusing membranes are plotted against (B) a series of SNARE/membrane distances and (C) different membrane lipid compositions. Arrow in (B) indicates the most physiologically relevant distance when the closest points between SNARE and membranes are 3 Å, the thickness of a layer of water.[19] V (circles): Interaction energies between the SNARE complex and the v-membrane. T (squares): Interaction energies between SNARE and the t-membrane. VT (triangles): Interaction energies between the v- and the t-membranes if the SNARE complex were extracted.

Charged residues are evolutionarily conserved on the SNARE complex (Fig. 1C). A similar overall pattern of charge distribution is observed in endosomal SNARE complex (Fig. 4A). Moreover, the electrostatic interactions between endosomal SNARE and v- as well as t-membranes are similar to the neuronal SNARE (Fig. 4B, 4C). Hence, the role played by electrostatics during SNARE-mediated membrane fusion is likely to be evolutionarily conserved.

### Several SNARE Complexes Could Cooperatively Promote Bending of Fusing Membranes

**Figure 5:**
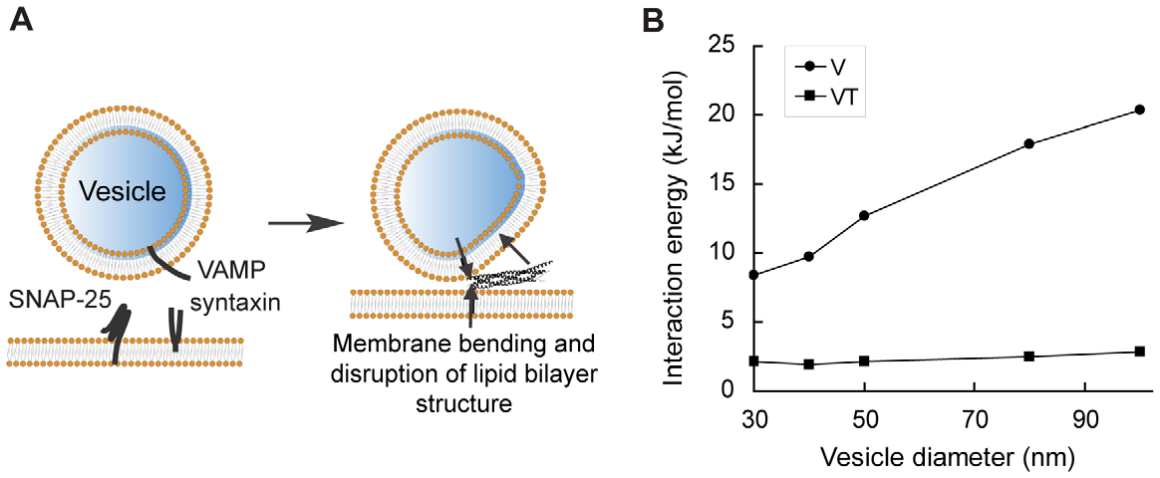
Estimate of membrane bending induced by electrostatics of SNARE complex. (A) Schematic diagram illustrating that free energy released in SNARE complex assembly is transmitted to the transmembrane domains and forces the closing of two fusing membranes (pair of vertical black arrows); meanwhile, strong electrostatic repulsion between SNARE and the v-membrane pushes the membrane away from the center of fusion (tilted black arrow). The resulting force couple should in principle promote bending of the v-membrane. Since SNARE/t-membrane electrostatic interactions are weak, no strong bending is expected on the t-membrane. (B) A standard curve for estimating the degree of bending induced by the electrostatics of SNARE. Interaction free energies between SNARE and a series of v-membranes that bear different spontaneous curvatures (i.e. local radii) were calculated.

Our calculations show that at the final stage of fusion, the v-membrane is electrostatically repelled from the site of fusion by the SNARE complex. Meanwhile, the v-membrane is also pulled toward the site of fusion by the SNARE TMDs and by local electrostatic attractions from positive charges at the SNARE core domain C-terminus (Fig. 5A). The discovery of this parallel but opposing force couple led us to ask whether membrane bending, crucial for fusion, can be generated or enhanced by this force couple. In support of this hypothesis, free energy required for membrane bending is estimated to be ∼40 kJ/mol,[37] the same order of magnitude as calculated electrostatic repulsion between SNARE and the v-membrane.

As a first step towards answering this question, we provided an estimate of the level of potential membrane bending generated by this force couple. Since the repulsion between SNARE and v-membrane is weaker for vesicles with smaller diameters which by definition have greater membrane bending, we asked: at what point will the energy penalty of membrane bending just be offset by a reduction in electrostatic repulsion exerted by SNARE complex as a result of greater bending?

From Helfrich's membrane elasticity theory[41], we can show that the bending energy penalty (see details of derivation in Methods):
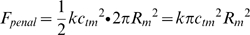
where 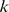 is bending rigidity, 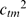 is the mean local curvature deviation, 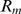 is the radius of vesicle. Since this penalty has to be less or equal to the energy compensation derived from reduction in SNARE/v-membrane repulsion when local bending occurs, we have
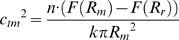
where 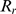 is the new local radius after bending takes place, and 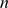 is the number of SNARE complexes acting at the same time.

Next, we systematically derived interaction energies between SNARE complex and v-membranes that bear a series of different curvatures as the “standard curve” (Fig. 5B). By plugging these values into the above equation, we found that on a typical synaptic vesicle of 40 nm-diameter, 5 SNARE complexes acting in synergy will produce at least a 25% reduction in local diameter.

We argue that this rough estimate is an underestimate because: (1) In our calculations we assumed that the entire lower hemisphere of synaptic vesicles is bended to the same degree. However, in reality the effect is necessarily more local (Fig. 1A); (2) Thermal fluctuation will cause lateral rotation of the SNARE complex and thus transient increase in electrostatic repulsion (Fig. 2B-2D) and transient increase in bending; (3) Binding of auxiliary factors to SNARE could also enhance the level of bending (see below).

### Effects of Complexin or Synaptotagmin Binding to the SNARE Complex

Complexin and synaptotagmin are important auxiliary proteins to the SNARE complex.[6], [7] They can directly bind to the SNARE complex; but mechanistically, how they influence the course of membrane fusion is not well understood, particularly for complexin.[6], [42] We therefore tested whether their association with the SNARE complex results in any changes in SNARE/membrane electrostatic interactions.

**Figure 6:**
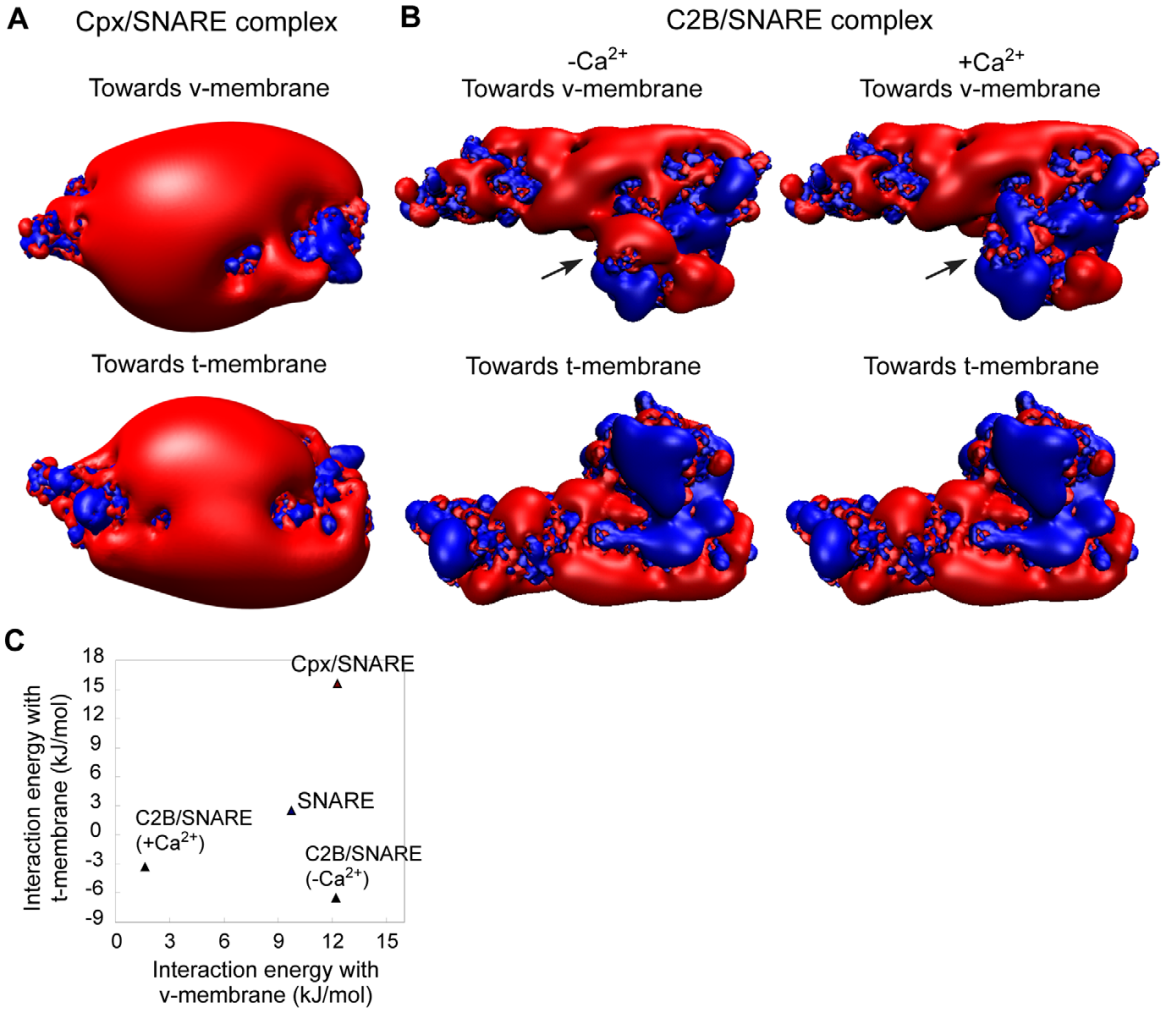
Complexin and synaptotagmin modulate electrostatic interactions between SNARE complex and membranes. (A) Surface isopotential contours of the complexin/SNARE complex rendered at ±1 kT/e. (B) Surface isopotential contours at ±1 kT/e for the C2B/SNARE complex, with or without Ca^2+^ ions bound to the Ca^2+^-binding loops of the C2B domain. Positive potential is colored in blue, and negative potential is in red. (C) An energy landscape of electrostatic interactions between SNARE complex and membranes as it associates and dissociates with complexin and synaptotagmin.

We first plotted the surface potential profile of the complexin/SNARE (cpx/SNARE) complex[23] (Fig. 6A). Intriguingly, although complexin binds to the v-membrane-facing side of SNARE, association with complexin results in dramatic expansion of negative potential on the t-membrane-facing side of the SNARE complex (compare Fig. 6A with Fig. 1B). Calculations showed that, in the presence of complexin, the SNARE complex electrostatically repels both v- and t-membranes (∼12 kJ/mol and ∼15 kJ/mol, respectively; see Fig. 6C). In principle, this could promote bending of both membranes to a degree greater than SNARE complex alone. Indeed, applying similar analyses to estimate the degree of membrane bending, we found that the difference in interaction energies between cpx/SNARE complex and v-membranes of 40 and 30 nm-diameter, is 16.4 kJ/mol−14.6 kJ/mol = 1.8 kJ/mol. This is greater than the SNARE complex without complexin (1.35 kJ/mol).

Next, we addressed the electrostatics of synaptotagmin. Synaptotagmin is the putative Ca^2+^ sensor during synaptic vesicle release, and has been shown to bind to both membrane and the SNARE complex.[31], [32] Because no crystal structure is available for the synaptotagmin/SNARE (syt/SNARE) complex, we utilized a structure based on molecular docking, NMR analyses in solution and other experimental data.[9] In this structure, only the C2B domain is present and bound to the SNARE complex. The C2A domain does not bind to the SNARE complex and is involved in direct insertion into lipid bilayers.[7] Although Ca^2+^ greatly enhances synaptotagmin binding to SNARE, it is unclear whether synaptotagmin is bound to the SNARE complex before Ca^2+^ influx in neurons. We thus analyzed the syt/SNARE complex with and without bound Ca^2+^ ions separately.

First, surface potential profiles showed that there are significantly more positive charges in the t-membrane-facing side of the C2B/SNARE complex compared to SNARE alone, regardless of whether Ca^2+^ is bound to C2B (Fig. 6B). The presence of Ca^2+^ mainly affects the electrostatic pattern of the v-membrane-facing side. Whereas Ca^2+^-free C2B/SNARE exhibits a mixture of positive and negative charges on this side, Ca^2+^ conferred a cluster of positive charges when bound to C2B (Fig. 6B). Quantitative analyses revealed an interesting trajectory on the energy landscape as SNARE associates and dissociates with complexin and synaptotagmin (Fig. 6C). According to these results, binding of Ca^2+^-free synaptotagmin to SNARE complex turns SNARE's interaction energy with the t-membrane from near-neutral to attractive. Upon Ca^2+^ binding, the existing repulsion between SNARE and the v-membrane further disappears. Synaptotagmin therefore presumably allows for stepwise close apposition of SNAREs and fusing membranes.

The unique electrostatics of synaptotagmin may have important roles that extend beyond the fusion-initiation stage. In principle, the C2B/SNARE complex could promote membrane bending by a mechanism different from the one depicted in Fig. 6 for SNARE complex or cpx/SNARE complex. Attractive electrostatic interactions between C2B/SNARE and the membranes could maintain membrane bending after fusion pore opening by bending fusing membranes inwards.[43] Moreover, with intact C2A domains, synaptotagmin alone may be sufficient to induce membrane bending by membrane insertion.[44]

## Discussion

Using molecular mechanics simulations, we performed a systematic theoretical analysis of electrostatic interactions in SNARE-mediated membrane fusion. Our findings suggest that surface charges of SNARE complex are conserved and strategically placed so that a highly energetically favorable and structurally preferred orientation exists for SNARE complexes in between fusing membranes. Such a preferred orientation prompted us to propose a “propeller” mechanism for SNAREs, in which synaptotagmin binding and membrane insertion drive SNARE complex to laterally turn over by 180°, causing SNARE TMDs to disrupt lipid bilayer structures. We further predicted that as SNARE complex associates and dissociates with complexin and synaptotagmin, a series of membrane bending events may occur which sequentially set the stage for rapid and tightly controlled release of neurotransmitters at synapses.

### Preferred Lateral Orientation of SNARE Complex between Fusing Membranes

We propose that we identified a preferred relative rotation for SNARE-based fusion machineries between fusing membranes. This is supported both by structural analyses and by energetic minima at preferred orientation for not only the SNARE complex, but also the cpx/SNARE complex and the syt/SNARE complex. In all three cases, deviation away from the same preferred rotation is accompanied by dramatic increase in electrostatic repulsion from the neighboring membranes at a magnitude enough to tweak the orientation of SNARE complex back to the resting position. Hence, surface electrostatic potential of both the SNARE proteins and their regulatory factors appears to have been evolutionarily optimized for this structurally preferred rotation.

### A Hypothetical “Propeller” Mechanism of SNARE-Driven Membrane Fusoin

The existence of preferred rotation for SNARE complex between membranes led us to an intriguing novel hypothesis of SNARE-driven membrane fusion. According to the preferred rotation we identified, the Ca^2+^-binding loop of the synaptotagmin C2B domain points straight toward the v-membrane in the C2B/SNARE complex.[9] The Ca^2+^-binding loop is the structure that inserts into the membrane in response to the Ca^2+^ signal. Hence, if C2B preferentially inserts into the PIP_2_ domains of t-membrane as previously suggested[34], [35], [36], it should turn over by 180° to target the t-membrane in response to Ca^2+^. During this process, Ca^2+^-trigger membrane penetration of synaptotagmin should steer the SNARE complex, on which it is bound, to also rotate laterally by 180° round its longitudinal axis away from the preferred relative rotation. Because SNARE complex are coupled to the membranes by two TMDs and a palmitoylated anchor, turning over of the SNARE complex should cause the membrane anchors to drastically stir and disrupt local membrane structure, perhaps causing fusion pore opening.

This model enables a powerful Ca^2+^-coupled step with kinetics essentially equal to synaptotagmin membrane insertion. Remarkably, both C2A and C2B domains are able to penetrate the membrane with nanomolar affinities, which is a binding energy (∼50 kJ/mol) sufficient for overcoming the energy barrier to lateral rotation of SNARE complex (Fig. 2B). Further studies are needed to explore this hypothesis experimentally.

### Implication for the Mechanisms of Function of Complexin and Synaptotagmin

How complexin plays its critical role in calcium responsiveness is poorly understood, in part due to its very simple helical structure. We propose that one possible mechanism is electrostatic. Our findings predict that it catalyzes a transition from unilateral membrane bending to bilateral bending. Bilateral bending is a high-energy state and may represent the mechanistic underpinning of the proposed metastable state[28] associated with the complexin/SNARE complex.

Complexin and synaptotagmin are mutually exclusive in binding to the SNARE complex. Since loss-of-function data show that they are both essential for Ca^2+^-responsiveness in synaptic exocytosis, their sequence of binding and functional relationship remain unclear. Our findings suggest that synaptotagmin could abolish the repulsions between SNARE and membranes after calcium influx at synapses. In addition, several studies suggest that synaptotagmin may mediate membrane bending on its own (e.g. refs.[43], [44]). Together, synaptotagmin may not only facilitate membrane contact through electrostatic mechanisms, but also maintains membrane bending by electrostatic-independent means. On the other hand, complexin binding to SNARE complex could induce bilateral membrane bending, which represents a high-energy state and a less stable intermediate. Since physical contact between membranes is only the first step in fusion while membrane bending has to be maintained throughout the fusion process,[26], [45], [46] it is possible that synaptotagmin binding precedes complexin. Alternatively, the two binding events are not necessarily sequential. In principle, the several SNARE complexes present at a single fusion site could have different states of complexin- or synaptotagmin-binding. In this way, a balance may be achieved between a lower electrostatic energy barrier and a sufficient level of membrane bending.

## Methods

### Models for Membranes and Proteins

Atomic-detail models of lipid bilayes were constructed from models for palmitoyl-oleyl-phosphatidylserine (POPS) and palmitoyl-oleyl-phosphatidylcholine (POPC) as previously described.[15], [17], [18], [19] The model t-membrane is planar and consists of 528 lipids, with a dimension of ∼200 Å×100 Å×60 Å. The v-membrane consists of 352 lipids, with a dimension of ∼135 Å×100 Å×60 Å. V-membranes are curved to various degrees to the spontaneous curvatures of synaptic vesicles and dense-core vesicles. These membranes were sufficiently large so that they extend at least 4 Debye lengths beyond the boundary of SNARE complex in all directions.

Models of proteins include: neuronal SNARE core complex (PDB entry 1SFC), complexin/SNARE complex (PDB entry 1KIL), endosomal SNARE core complex (PDB entry 1GL2), and synaptotagmin/SNARE complex.[9], [10] Hydrogen atoms were added to the heavy atoms by PDB2PQR.[47] Partial atomic charges and atomic radii were assigned to each atom using a CHARMM parameter set.

Models for the SNARE/v-/t-membrane ternary complex were built according to current knowledge of the fusion-competent state, when trans-SNARE complex assembly was just complete (Fig. 3A). We performed extensive planar translations of SNARE across the membranes to ensure that the interaction energies are not sensitive to position of SNARE complex with respect to t-membrane (data not shown); we also tested different SNARE N-terminal elevations, and in all cases the same conclusions on the nature of electrostatic interactions were reached. For the C2B/SNARE complex, we adopted the N-terminal elevation with lowest total system energy.

The relative lateral rotation of SNARE complex with respect to v- and t-membranes is based on considerations detailed in Results. Specifically, we placed the Lys93 of VAMP in neuronal SNARE complex closest to the v-membrane, and Ser259 of syntaxin closest to the t-membrane. The same procedures were applied to the endosomal SNARE complex to determine its relative orientation. The orientations for complexin/SNARE and C2B/SNARE complexes were determined by aligning them with the neuronal SNARE complex.

The distance between proteins and membranes in this study is defined as the nearest vertical distance between the van der Waals surfaces of two macromolecules.[18] Except otherwise mentioned, the default distance is 3 Å,[19] and the default lipid composition for model membranes is 2∶1 PC/PS.

### Molecular Dynamics

Molecular dynamics simulations were performed with GROMACS[48] simulation package (version 3.3.1) and OPLS-AA/L all-atom force field. For neuronal SNARE complex, residues in the linker regions that bridge the core complex and the TMDs which are absent from the crystal structure were added back in α-helix configuration. The resulting structure, together with 18807 SPC water molecules, was placed in a 16 nm×5.5 nm×7 nm box. All molecular dynamics simulations were performed with NPT ensemble at 300 k temperature and 1 bar pressure. After energy minimization and equilibration runs, 5 cyclic runs were conducted, each including a stretching process with AFM pulling, a 1 ns equilibration run and a 200 ps production run. C-terminal residues of VAMP and synatxin were pulled apart toward the t- and v-membranes, respectively, with a maximum velocity of 0.0025 nm/ps, with strong spring, to simulate the trans-SNARE complex. Next, equilibration runs were performed to remove occasional irregular angles in the structure. The resulting structure was then recorded every 5 ps, and was aligned and averaged for electrostatic calculations. SNARE complex C-termini were intended to be pulled apart by 5, 10, 20 and 30 Å, respectively. The final terminal distance increments between VAMP and syntaxin were 4.812 Å, 9.651 Å, 20.250 Å and 30.048 Å.

### Numerical Solution of the Nonlinear Poisson-Boltzmann Equation

The nonlinear Poisson-Boltzmann equation was solved numerically with parallel multigrid focusing, using the Adaptive Poisson-Boltzmann Solver (APBS).[49] Solvent was represented implicitly as a homogenous dielectric medium, and macromolecules were modeled to atomic detail. Nonlinear PBE was first solved on a 417×225×417 coarse grid about 7 times larger than the size of macromolecular models in all axes, with single Debye-Huckle boundary conditions, and then on a fine grid, with the same number of grid points, that just spans the system under investigation. The final grid resolution was ∼0.5 Å. All calculations were performed at physiological ionic strength (135 mM KCl). The dielectric constant of regions inside the macromolecules was 2, and was 78.54 for the solvent. Solvent radius was specified to that of water, i.e. 1.4 Å.

The precision of calculations was estimated by two means. First, we used a lower focusing grid resolution (1 Å/grid instead of 0.5 Å/grid) to perform the same interaction energy calculations.[18] Second, we doubled the grid lattice size to >14 times the dimension of the model for macromolecule complexes in all axes. Both methods yielded errors <0.8 kJ/mol.

### Estimate of Membrane Bending Induced by Electrostatics of SNARE Complex

From Helfrich's membrane elasticity theory,[41] bending free energy density of biological membrane satisfies the following equation under harmonic approximation,
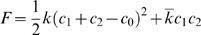
where 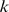, 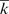 and 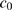 are bending rigidity, Gaussian bending constant and spontaneous curvature, respectively. The bending free energy of a vesicle equals the integration of the free energy density along its surface. For a spherical vesicle, the parameters satisfy the following equations under the condition of energy density minimization,
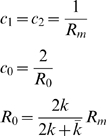
where 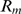 is the radius of vesicle, and 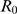 is spontaneous radius directly related to spontaneous curvature 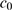. Applying Gauss-Bonnet theorem, the Gaussian curvature term does not change and need not be considered as long as the vesicle is integral. Thus we have
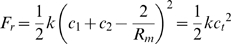
Here, vesicle radius 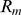 replaces spontaneous radius 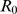 for simplicity. 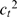 is a measure of local curvature deviation. Hence, the curvature energy of a vesicle is minimal when its shape is exactly spherical, and any deviation from that suffers energy penalty. Safely assuming that repulsion only influences the lower hemisphere of a vesicle (because Debye length in physiological ionic strength is only ∼1 nm), we have the bending energy penalty
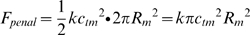
where 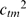 is the mean local curvature deviation. Since this penalty has to be less or equal to the energy compensation derived from reduction in SNARE/membrane repulsion when local bending occurs, we have
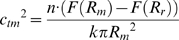
where 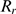 is the new local radius after bending takes place, and 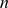 is the number of SNARE complexes acting at the same time.
